# Supplementary figures and images for: Axonal growth on surfaces with periodic geometrical patterns
Source: PLoS One. 2021 Sep 23;16(9):e0257659. doi: 10.1371/journal.pone.0257659 (PMC8459970; doi:10.1371/journal.pone.0257659)

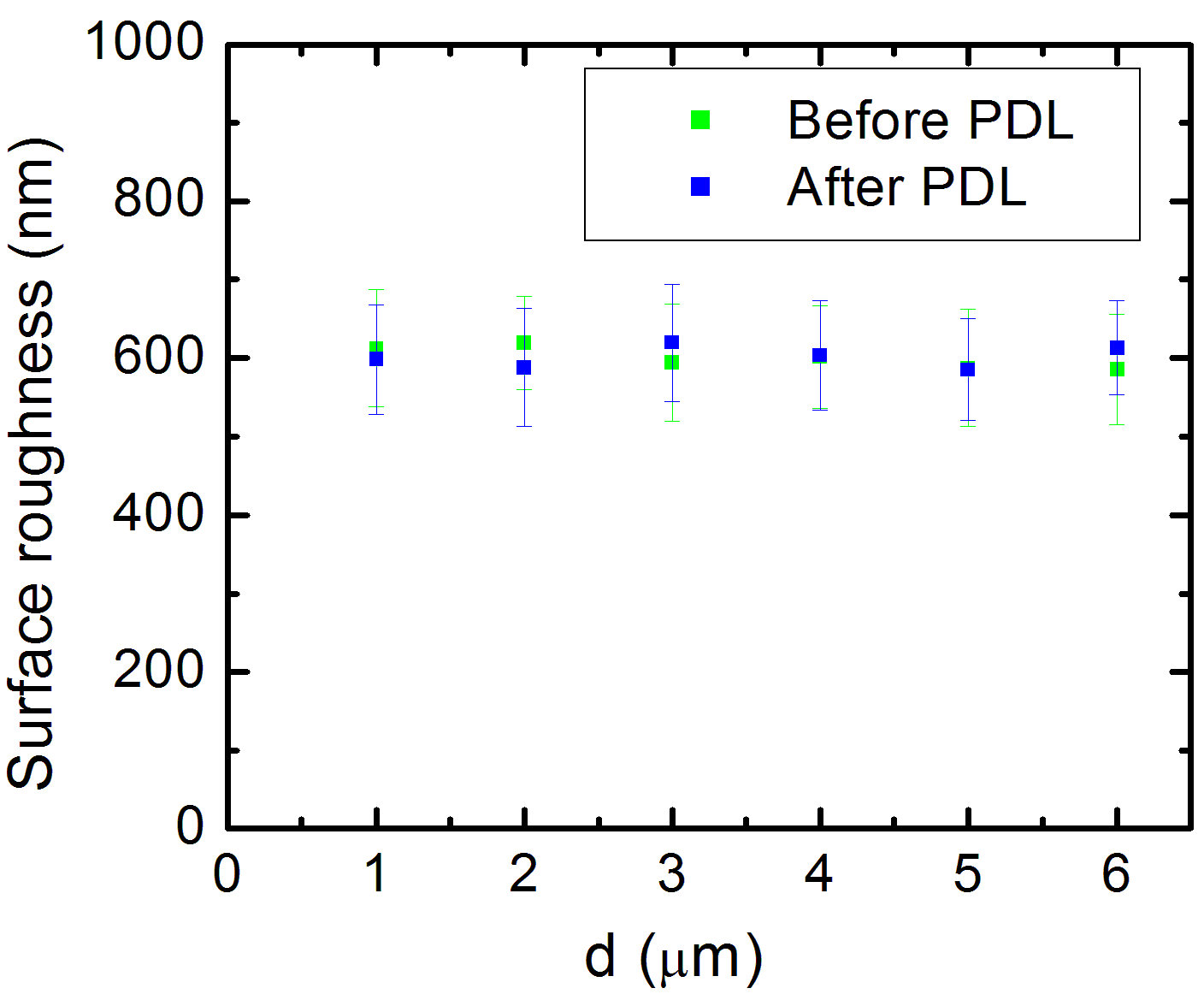

Supplement: S1 Fig — The surface roughness is measured with the AFM. The green data points show the average surface roughness measured before PDL coating. The blue data points show the average surface roughness measured on the same surfaces after coating with PDL. The error bars indicate the standard error of the mean. The data demonstrates that the surface roughness does not vary significantly among the PDMS surfaces with different spatial periods d, and it does not change significantly upon surface coating with PDL. The variation of the average roughness among these substrates is less than 10%. (TIF) [file pone.0257659.s001.TIF]

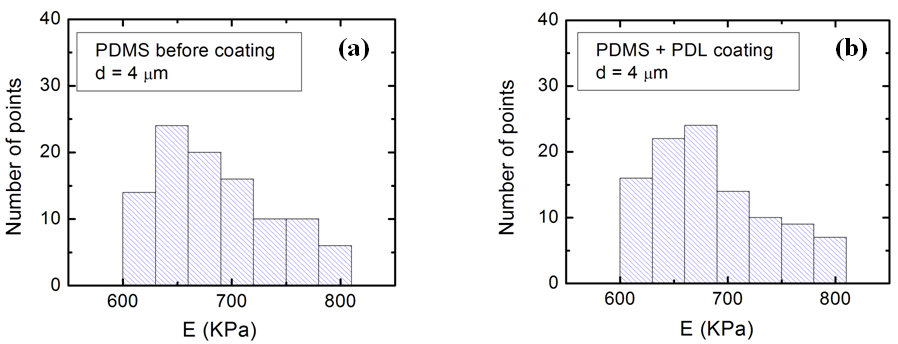

Supplement: S2 Fig — (a) Histogram for elastic modulus for a PDMS surface with d = 4 μm, measured before coating the surface with PDL. (b) Histogram for elastic modulus for the same PDMS surface shown in (a), measured after coating the surface with PDL. The two maps display similar ranges for E. The average elastic modulus between the two maps differs by only 5%. The data demonstrates that PDL coating does not change the elastic modulus of the PDMS substrate. (TIF) [file pone.0257659.s002.tif]

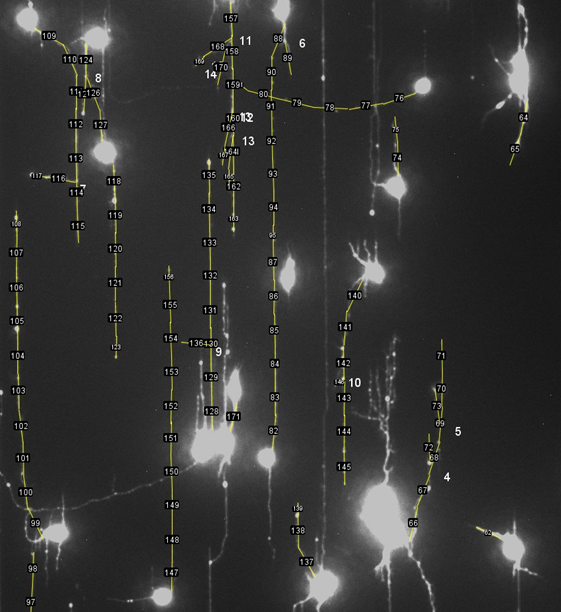

Supplement: S3 Fig — The segments marked in yellow are superimposed on the axons and show the growth trajectory. The numbers on each segment represent different positions of the growth cone during growth. Each segment is 20 μm in length as described in the Data Analysis section in the main text. (TIF) [file pone.0257659.s003.tif]

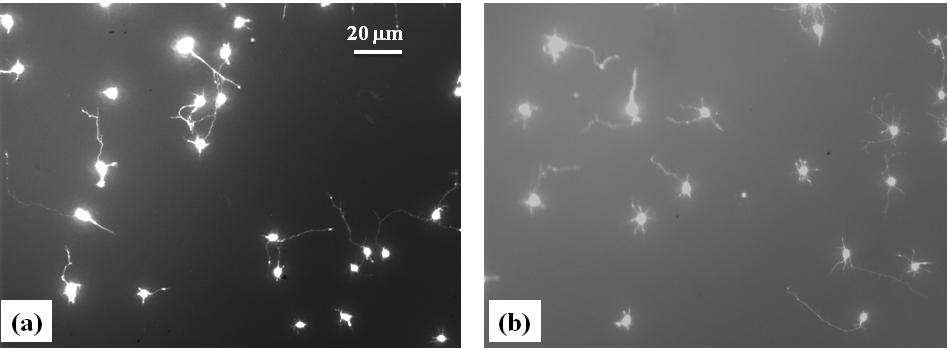

Supplement: S4 Fig — Fluorescence images showing examples of axonal growth for cortical neurons treated with Taxol (a) and Blebbistatin (b). The images are captured 42 hrs after neuron plating. The scale bar shown in (a) is the same for both images. The pattern spatial period is d = 5 μm for both images. (TIF) [file pone.0257659.s004.tif]

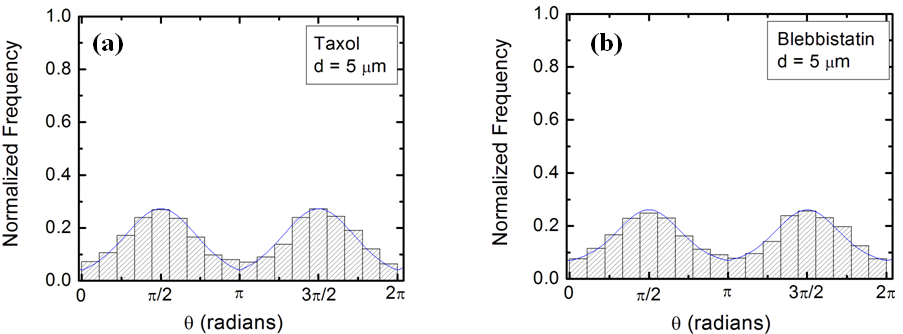

Supplement: S5 Fig — Normalized experimental angular distributions for axonal growth measured for neurons treated with Taxol (a) or Blebbistatin (b). The angular distributions are measured for cell cultured on micropatterned PDMS surfaces with pattern spatial period is d = 5 μm. The vertical axis (labeled Normalized Frequency) represents the ratio between the number of axonal segments growing in a given direction and the total number N of axon segments. Each axonal segment is of 20 μm in length (see section on Data Analysis in the main text). All distributions show data collected at t = 42 hrs after neuron plating. (a) Angular distribution obtained for N = 734 different axon segments for neurons treated with Taxol. (b) Angular distribution obtained for N = 694 different axon segments for neurons treated with Blebbistatin. The neurons treated with either Taxol or Blebbistatin show a significant decrease in the degree of alignment with the surface patterns, compared to the untreated cells. The continuous blue curves in each figure are the predictions of the theoretical model presented in the main text. (TIF) [file pone.0257659.s005.tif]

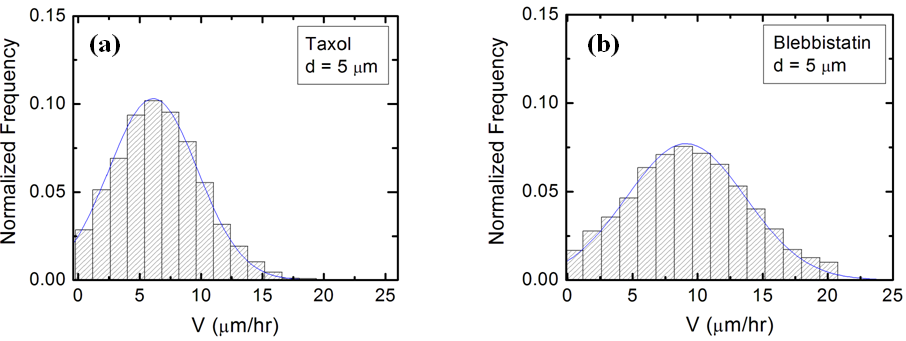

Supplement: S6 Fig — Normalized speed distributions obtained for growth cones of cortical neurons treated with Taxol (a) and Blebbistatin (b). The growth substrates are PDL coated PDMS surfaces with periodic micropatterns with the pattern spatial period d = 5 μm. (a) Speed distribution measured for N = 221 different growth cones for neurons treated with Taxol. (b) Speed distribution measured for N = 206 different growth cones for neurons treated with Blebbistatin. The continuous blue curves in each figure are the predictions of the theoretical model presented in the main text. (TIF) [file pone.0257659.s006.tif]

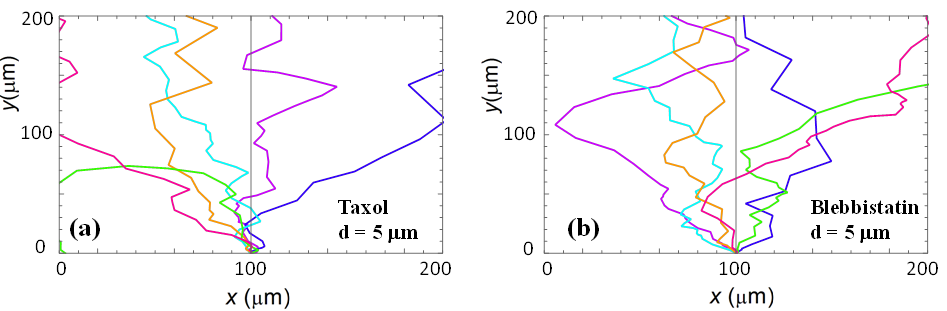

Supplement: S7 Fig — Examples of simulated neuronal growth for neurons treated with Taxol (a) or Blebbistatin (b). The simulations are performed by using the values of the growth parameters obtained from the fit of the experimental data with Eqs 2 and 4 (seen main text). The pattern spatial period for both images is d = 5 μm, corresponding to the experimental data shown in S4–S6 Figs. (TIF) [file pone.0257659.s007.tif]
