# Supplementary material for: Axonal growth on surfaces with periodic geometrical patterns
Source: PLoS One. 2021 Sep 23;16(9):e0257659. doi: 10.1371/journal.pone.0257659 (PMC8459970; doi:10.1371/journal.pone.0257659)
Supplement: S1 Table — The guiding parameter decreases with increases pattern curvature radius R, as well as upon chemically treatment of neurons. All measurements are performed at the same time (t = 42 hrs after plating). (PDF) [file pone.0257659.s008.pdf]

**Cell/Substrate**	γ_θ / D_θ
Untreated/ PDMS R =*0.5* μm	8.9 ± 0.9
Untreated/ PDMS R=*0.7* μm	4.6 ± 0.8
Untreated/PDMS R=*1* μm	2.6 ± 0.8
Untreated/PDMS flat substrate (ref. [20, 33])	0.6 ± 0.8
Untreated/Glass flat substrate (ref. [19, 20])	0.5 ± 0.9
Blebbistatin/PDMS R=*0.5* μm	3.2 ± 0.9
Blebbistatin/PDMS R=*1* μm	1.1 ± 0.8
Taxol/PDMS R=*0.5* μm	4.7 ± 0.9
Taxol/PDMS R=*1* μm	1.2 ± 0.8
Untreated/nanoppx R=*0.5* μm (ref. [10])	6.0 ± 1
